# Supplementary material for: Role of Human Microbiome in Development and Management of Head and Neck Squamous Cell Carcinoma
Source: Cancers (Basel). 2025 Jul 3;17(13):2238. doi: 10.3390/cancers17132238 (PMC12249478; doi:10.3390/cancers17132238)
Supplement: Supplementary file 1 [file cancers-17-02238-s001.zip › cancers-3683350-supplementary.pdf]

S1: Table 1: Influence of gut microbiome on systemic anti-cancer therapy

Samples:	Results / implications	Ref.
**1) Chemotherapy (CHT)**		
**Mouse model**	Mice pretreated with antibiotics (ATB; vancomycin, imipenem, and neomycin) exhibited lower response rate to platinum-based CHT across several cancer types	Iida *et al.* (2013) [137]
**Mouse model**	ATB therapy (ampicillin, neomycin, metronidazole, and vancomycin) accelerated tumor growth and decreased response rate to platinum-based CHT in epithelial ovarian cancer mouse model	Chambers *et al.* (2022) [139]
**Mouse model**	cDDP and 5-FU combination CHT does not affect gut microbiome bacterial diversity, however, it increases diversity of esophageal microbiome; medicinal botanicals, *e.g. Actinidia polygama* have anti-cancer and anti-metastatic effects	Cheung *et al.* (2020) [140]
**Mouse model**	*A. muciniphila* with cDDP exhibited better anti-cancer effect than cDDP alone in lung cancer model	Chen *et al.* (2020) [142]
**Mouse model**	CBDCA induces gut mucositis in mice; abundance of *P. copri* rose throughout CHT treatment – the amount of *P. copri* correlated with gut mucositis severity; mice with reduced abundance of *P. copri* (by metronidazol pre-treatment) had attenuated incidence and severity of gut mucositis	Yu *et al.* (2019) [144]
**Mouse model**	Mice treated concurrently with 5-FU and combination of ATB (vancomycin, ampicillin, and neomycin) had much worse treatment response compare to mice treated only with 5-FU; use of probiotics had no improvement in response rate	Yuan *et al.* (2018) [145]
**Mouse model**	*F. nucleatum* increases chemoresistance to 5-FU in colorectal cancer by modulating autophagy	Yu *et al.* (2017) [146]
**2) Immunotherapy**		
**Stool from metastatic melanoma patients, subsequently tested in germ free mice and mice after FMT**	Composition of commensal microbiota is associated with immunotherapy response; *B. longum* was found to be associated with improved treatment response to ICIs; host microbiome enriched with *Ent. faecium*, *Collinsella aerofaciens*, *B. adolescentis*, and *Parabacteroides merdae* were associated with worse treatment outcome; mice who received FMT from ICIs-responder had better anti-cancer results compared to those who received FMT from ICIs-non-responder	Matson *et al.* (2018) [172]
**Gut and/or oral microbiome of metastatic melanoma patients**	Gut microbiome modulates response to ICIs in melanoma patients; microbiome of high diversity and/or increased abundance of family *Ruminococcaceae*/genus *Faecalibacterium* respond better to ICIs treatment; microbiome of low diversity	Gopalakrishnan *et al.* (2018) [173]

	and/or high relative abundance of Bacteroidales is associated with worse response to ICIs	
**Fecal samples of 39 metastatic melanoma patients**	Responders to all ICIs (ipilimumab, nivolumab, their combination, pembrolizumab) had microbiome with increased abundance of *Bact. caccae*; increased representation of *Faecalibacterium prausnitzii*, *Bacteroides thetaiotamicron*, and *Holdemania filiformis* was reported among responders to ipilimumab and nivolumab combination; abundance *Dorea formicogenerans* was enriched in microbiome of pembrolizumab responders; in metabolomic assays high levels of anacardic acid was found in ICIs responders	Frankel *et al.* (2017) [174]
**Fecal samples of 26 metastatic melanoma patients treated with ipilimumab**	Patients with dominant phylum Firmicutes, *e.g.* genus *Faecalibacterium*, had longer PFS and OS, compared to patients with dominant *Bacteroides* in baseline microbiome; all cases of immune-related colitis occurred in the group with dominant phylum *Firmicutes*, *e.g.* relatives of *Faecalibacterium prausnitzii* and *Gemmiger formicilis*	Chaput *et al.* (2017) [175]
**Fecal samples of 27 metastatic melanoma patients**	Higher microbial diversity is associated with longer PFS; cluster analysis divided patients into three groups: low-risk (highly abundant *Faecalibacterium prausnitzii*, *Coprococcus eutactus*, *P. stercorea*, *S. sanguinis*, *S. anginosus*, and *Lachnospiraceae* bacterium strain 3 1 46FAA) group had 99% lower chance of developing prognosis compared to high-risk group (highly abundant *Bact. ovatus*, *Bact. dorei*, *Bact. massiliensis*, *R. gnavus*, and *Blautia producta*)	Peters *et al.* (2019) [176]
**Fecal samples of 165 metastatic melanoma patients**	Confirmed link between microbiome and PFS and OS; increased abundance *B. pseudocatenulatum*, *Roseburia* spp., *Phascolarctobacterium succinatutens*, *Lim. vaginalis*, and *A muciniphila* were associated with responded to ICIs; microbiome enriched with *B. clarus* was associated with non-responders to ICIs	Lee *et al.* (2022) [177]
**Fecal samples of 94 melanoma patients**	Specific taxa were associated with non-progressors (*R. torques*, *B. producta*, *B. wexlerae*, *B. hansenii*, *Eu. rectale*, *R. gnavus*, and *Anaerostipes hadrus*) and progressors (higher abundance of *Prevotella* spp., *Oscillibacter* spp., *Alistipes* spp., and *Sutterellaceae*) on ICIs treatment	McCullch *et al.* (2022) [178]
**Fecal samples of 338 NSCLC patients**	Objective response rate to ICIs (nivolumab, atezolizumab, pembrolizumab) was 28% *vs.* 18% for patients with present and absent *A. muciniphila*, respectively	Derosa *et al.* (2022) (179)
**Mouse melanoma model treated with ipilimumab**	CTLA-4 blockade depends on *Bacteroides* spp. representation; high abundance of *Bact. thetaiotaomicron* and *Bact. fragilis* was linked to higher efficacy of CTLA-4 inhibitors; mice pre-treated by antibiotics and germ-free mice were non-responding to CTLA-4 inhibitors; FMT with *Bact. fragilis* from humans to mice overcame non-responding phenotype	Vétizou *et al.* (2015) [180]
**Animal models of colorectal and bladder cancer, and melanoma**	*B. pseudolongum*, *L. johnsonii*, and *Olsenella* spp. significantly enhance ICIs efficacy through increased translocation of inosine and activation of CD8+ T lymphocytes	Mager *et al.* (2020) [181]

**Mouse melanoma model**	*Bifidobacterium* spp. is associated with enhanced antitumor effects; in combination with ICIs they significantly inhibit tumor growth	Sivan *et al.* (2015) [182]
**128 cancer patients, majority of them were treated for metastatic melanoma**	Commercial probiotics do not improve ICIs response rate; diet rich in fiber was associated with significantly prolonged PFS	Spencer *et al.* (2021) [183]
**Fecal samples of metastatic melanoma patients**	*Bacteroides* phylum is highly abundant in patients who do not develop immune-related colitis	Dubin *et al.* (2016) [186]

S2: Table 2: Microbial alterations discovered in HNSCC patients

No. of patients	Sample	Method	Results / implications	Ref.
**N/A**	N/A	Metanalysis	Significant association between periodontitis and presence of cancer, including oral cancer	Corbella *et al.* (2018) [3]
**N/A**	N/A	Review	Significant association between periodontitis and presence of cancer, especially significant in oral and esophageal cancer	Fitzpatrick *et al.* (2010) [7]
**N/A**	N/A	Metaanalysis	Association between periodontal disease and oral cancer, in some studies attenuated if adjusted for tobacco and alcohol consumption	Javed *et al.* (2016) [8]
**42 OSCC patients**	Buccal swabs	16S rRNA sequencing	Changes in bacterial diversity and microbial representation, enrichment of the genus *Fusobacterium*, loss of genus *Streptococcus* in OSCC patients	Su *et al.* (2021) [9]
**N/A**	N/A	Review	OSCC tissues were enriched with *F. nucleatum*, *P. intermedia*, *Aggregatibacter segnis*, *Capnocytophaga leadbetteri*, *Peptostreptococcus stomatis*, *Porphyromonas gingivalis*, and *Ps. aeruginosa*	Kakabadze *et al.* (2020) [10]
**N/A**	N/A	Metaanalysis	*Porph. gingivalis* could be associated with OSCC development, *P. gingivalis* may be involved in epithelial-mesenchymal transition, neoplastic proliferation, and/or tumor invasion promotion	Lafuente Ibáñez et a. (2020) [11]
**376 people: 127 healthy controls, 124 OPMD), 125 (OSCC)**	Unstimulated saliva	16S rRNA sequencing	Significant changes in microbial profiles between OPMD and OSCC patients	Burcher *et al.* (2022) [24]

**88 OSCC patients, 90 controls**	Unstimulated saliva	16S rRNA sequencing	Higher relative abundance of *P. tannerae*, *F. nucleatum* and *P. intermedia,* and lower relative abundance of *S. tigurinus* in OSCC patients	Hsiao *et al.* (2018) [34]
**39 tongue cancer patients**	Resected tumor and healthy tissue	16S rRNA sequencing	Positive association of Zygomycota, *Bacteroides* spp. (*e.g. P. gingivalis*), fusobacteria (*e.g. F. nucleatum*) with tongue cancer; depletion of *Lactobacillus* spp. can induce bacterial dysbiosis and pro-oncogenic environment; bacteria producing acetaldehyde, *e.g. Rothia* spp., *Streptococus* spp. and *Prevotella* spp., can be involved in OSCC development in chronic alcohol consumption	Mukherjee *et al.* (2017) [35]
**4 OSCC patients, 4 healthy controls**	Oral swabs	RNA sequencing	Distinct differences in microbial activity and representation in oral microbiome between OSCC patients and healthy controls; higher activity of fusobacteria, *Selenomonas* spp., *Capnocytophaga* spp., and genera *Dialister* and *Johnsonella* in cancer patients compared to healthy controls, *Bacillus* spp., *Porph. catoniae, Kingella denitricans, Capn. gingivalis, Neisseria elongata,* Bacterium MGEHA from the candidate division SR1, *Veillonella* sp. oral taxon 780, *Aggregatibacter segnis* and *S. downei* were more active in healthy controls	Yost *et al.* (2018) (36)

**43 HNSCC patients, 78 healthy controls**	Unstimulated saliva	16S rRNA sequencing	Significant differences between HNSCC and healthy oral microbiome, in HNSCC patients microbiome dominated Actinobacteria (*e.g. Actinomyces* spp.), Bacteroidetes (*e.g. Prevotella* spp., *Porphyromonas* spp.), Firmicutes (*e.g. Streptococcus* spp., *Veillonella* spp.), Fusobacteria (*e.g. Leptotrichia* spp., *Fusobacterium* spp.), and Proteobacteria (*e.g. Neisseria* spp.); increased abundance of *Lactobacillus* spp., *Mesorhizobium* spp., and *Ochrobactrum* spp., and reduced abundance of *Neisseria* spp., *Lautropia* spp., and *Phyllobacterium* spp. in HNSCC patients; tumor development is decreased in germ-free mice, but is promoted by oral gavage of OSCC-related microbiota in OSCC mouse model	Frank *et al.* (2022) [38]
**121 HNSCC patients**	Oral swabs	16S rRNA sequencing	No significant differences in microbial diversity between tumor and normal tissue; reduced representation of *Actinomyces* spp. In tumor tissue compared to the healthy controls; increased abundance of genus *Parvimonas* in HNSCC patients	Wang *et al.* (2017) [39]
**31 HNSCC patients and 11 healthy controls**	Stimulated saliva	16S rRNA sequencing	No significant difference in the microbial diversity between healthy controls and HNSCC patients; *Fusobacterium* spp. and *Veilonella* spp. were associated with healthy controls; CRT significantly affects microbial compositions; representation of	Kumpitsch *et al.* (2020) (45)

			Haemophilus spp., *Veillonella* spp., *Granulicatella* spp. was significantly reduced after CRT, however, abundance of *Lactobacillus* spp., *Scardovia* spp., *Acinetobacter* spp., and *Enterococcus* spp. decreased	
**3 OSCC patients, 2 matched controls**	Unstimulated saliva	Pyrosequencing	Certain bacterial alterations are specific to OSCC and may be sued for a diagnostic tool before development of invasive HNSCC	Pushalkar *et al.* (2011) [48]
**19 HNSCC patients, 25 healthy controls**	Tumor samples and salivary rinses	16S rRNA sequencing	Association of *Lactobacillus* spp., *Weeksellaceae* spp., *Veillonella* spp., *Megasphaera* spp. and family *Anaerolineae* with HPV-positive HNSCC; reduced abundance of *Haemopilus* spp., *Aggregatibacter* spp., and family *Gemellaceae*, and loss of *Prevotella* spp. representation in HNSCC patients; significantly higher abundance of *Neisseria* spp., *Eikinella* spp., and *Leptotrichia* spp. in HPV-negative HNSCC patients; significantly higher abundance of *Lactobacillus* spp., *Streptococcus* spp., *Staphylococcus* spp., and *Parvimonas* spp. in the HNSCC patients´ oral microbiome	Guerrero-Preston *et al.* (2016) [49]
**11 OSCC patients, 11 healthy controls**	Stimulated saliva	16S rRNA sequencing	Microbial profiles of OSCC patients and healthy profiles distinctly differ; slightly higher microbial diversity in OSCC saliva; higher relative abundance of phyla Bacteroidetes (*e.g. Prevotella* spp.), Proteobacteria (*e.g. Haemophilus* spp. and *Neisseria* spp.), and Firmicutes (*e.g. Streptococcus* spp. and *Veillonella* spp.) was	Wolf *et al.* (2017) [50]

			observed in healthy controls, higher abundance of phyla Actinobacteria (*Actinomyces* spp.), Firmicutes (*Schwartzia* spp., *Selenomonas* spp.), and Spirochaetes (*Treponema* spp.) was reported in OSCC patients	
**787 HNSCC patients, 537 saliva, mid-vagina, and vaginal introitus samples**	Saliva, mid-vagina, and vaginal introitus samples	16S rRNA sequencing	Identified commensal species from the vaginal microbiota, *Lim. johnsonii, L. gasseri* and *Lim. vaginalis*, in saliva of oropharyngeal cancer patients regardless of their HPV status - hypothesized that the presence is due to orogenital transmission during oral sex; identified higher abundance of *F. nucleatum* in HNSCC patients	Guerrero-Preston *et al.* (2017) [51]
**20 OSCC patients**	Tumor samples	16S rRNA sequencing	Identified certain taxa which were formerly only identified from environmental sources, *e.g. Dietzia psychralcaliphila* and *Gordonia sputi*; identified several human pathogens in the oral microbiome which could be associated with OSCC, *e.g.* genera *Micrococcus*, *Propionibacterium*, *Streptomyces*, *Bacillus*, *Enterococcus*, *Exiguobacterium*, *Staphylococcus*, and *Pseudomonas*; detected *S. anginosus* in the tumor tissue	Hooper *et al.* (2006) [53]
**20 OSCC patients, 20 healthy controls**	Tumor samples, deep epithelial swabs for healthy controls	16S rRNA sequencing	Phylum Fusobacteria and taxa *Campylobacter* spp. and *Pseudomonas* spp. were the most significantly associated with OSCC; *Streptococcus* spp., *Rothia* spp. and *Haemophillus* spp. were relatively more abundant in the healthy controls	Al-Hebshi *et al.* (2017) [54]

**122 004 prospectively examined participants, 129 HNSCC and 254 healthy controls**	Mouth washes	16S rRNA sequencing	*Corynebacterium* spp. and *Kingella* spp. were associated with decreased risk for HNSCC, significance tended to be larger for laryngeal cancer and tobacco users	Hayes *et al.* (2018) [55]
**68 HNSCC patients**	Tumor samples, oral rinses	16S rRNA sequencing	Phyla Firmicutes, Proteobacteria, and Bacteroidetes were predominantly present in HNSCC tissue; representation of *Fusobacterium* spp. was double as high as in healthy controls; *F. nucleatum* was associated with better prognosis in HNSCC, higher abundance of *Catonella* spp. and *Johnsonella* spp. was reported in non-smokers and non-drinkers; the stage of tumor positively correlated with amount of *Peptostreptococcus* spp.; decreased abundance of *Neisseria* spp., *Haemophilus* spp., and *Rothia* spp. was linked with worse cancer-specific survival	Chen *et al.* (2020) [60]
**212 HNSCC patients**	Tumor samples	16S rRNA sequencing	Significantly longer OS in *F. nucleatum*-positive tumors	Neuzillet *et al.* (2021) [61]

S3: Table 3: The effect of oral microbiota on treatment outcomes in HNSCC

Patients	Sample	Method	Results / implications	Ref.
**1) Radiotherapy (RT)**				
**68 HNSCC patients**	Unstimulated saliva, oral rinses	16S rRNA sequencing	RT decreases the numbers of neutrophils in the oral cavity, their counts do not return to the pre-RT values even 6 months after RT; RT decreases overall abundance of the present bacteria; RT significantly changes diversity of oral microbiota; increase in absolute abundance of *Streptococcus* spp. and *Lactobacillus* spp. was observed after RT; decrease in abundance of *Veillonella* spp., *Haemophilus* spp., *Neisseria* spp., *Actinomyces* spp., *Leptotrichia* spp., and *Capnocytophaga* spp. was observed during or after RT	Mojdami *et al.* (2022) [14]
**8 OSCC patients**	Supragingival plaque	Pyrosequencing, 16S rRNA sequencing	11 genera *Streptococcus, Actinomyces, Veillonella, Capnocytophaga, Derxia, Neisseria, Rothia, Prevotella, Granulicatella, Luteococcus*, and *Gemella* were identified as core-microbiota of HNSCC; relative abundance of major genera varied significantly without a significant trend	Hu *et al.* (2013) [15]

**19 HNSCC patients**	Unstimulated saliva, oral swabs	16S rRNA sequencing	Salivary microbiota does not significantly change during RT; HNSCC patients oral microbiome consistently contains dominantly *Streptococcus* spp., *Prevotella* spp., *Fusobacterium* spp. and *Granulicatella* spp.; Bacteroidales, *Capnocytophaga* spp., *Eikenella* spp., *Mycoplasma* spp., *Sneathia* spp., *Porphyromonas* spp., and *Tannerella* spp. are strongly associated with severe RIOM; pre-RT presence of *Fusobacterium* spp., *Haemophilus* spp., *Tannerella* spp., *Porphyromonas* spp., and *Eikenella* spp. correlated with higher incidence of severe RIOM	Vesty *et al.* (2019) [16]
**24 OSCC patients**	Unstimulated saliva	16S rRNA sequencing	Almost 30% of patients were scored with poor oral health, which is a strong predictor of development and severity of RIOM; at week 6 all patients scored at least Gr 1 RIOM, 50% and 42% of patients Gr 2 and Gr 3 respectively; in patients treated with CRT *Staphylococcus aureus*, *Ps. aeruginosa*, and *S. epidermidis* showed increased prevalence, while abundance of *Escherichia coli* decreased	Subramaniam *et al.* (2019) [106]
**28 HNSCC patients, healthy controls**	Supra- and subgingival biofilms, stimulated and unstimulated saliva	16S rRNA sequencing	90% of patients exhibited moderate or poor oral hygiene before initiation of RT; almost 60% of patients developed Gr 3 or 4 RIOM at time of completion of RT; abundance of *Actinomyces*	Gaetti-Jardim *et al.* (2018) [107]

			odontolyticus and *A. viscosus*, *Candida* spp., *Capnocytophaga ochracea*, *Eikinella corrodens*, *Enterococcus faecalis*, *Parvimona micra*, *Staph. intermedius*, *S. mitis* and *s. sobrinus* increased during RT, whereas the abundance of *A. naeslundii*, *Ps. aeruginosa*, *P. nigrescens*, and family *Enterobacteriaceae* rose particularly after RT; higher abundance of *Enterobacteriacae* family was associated with xerostomia	
**Unknown**	Oral rinses	Pyrosequencing, 16S rRNA sequencing	Throughout RT 16 phyla were identified, mostly members of phyla Actinobacteria, Bacteroidetes, Firmicutes, Fusobacteria, Proteobacteria, Spirochaetes, and Candidate Division TM7; five core genera were identified, *i.e. Actinomyces*, *Streptococcus*, *Veillonella*, *Campylobacter*, and *Prevotella*; richness and diversity of oral microbiome was reduced with increasing radiation dose, it was restored with time after RT	Gao *et al.* (2015) [108]

2) Chemotherapy (CHT)

**49 HNSCC patients**	Oral swabs, unstimulated saliva	16S rRNA sequencing	Oral mucositis is associated with 5-FU exposure; CHT decreases diversity of salivary microbial communities, such decrease correlates	Hong *et al.* (2019) [105]

			with the severity of oral mucositis; oral mucositis severity also correlates with increased abundance of *F. nucleatum* subsp. *vincentii*, Clostridiales, and *Treponema maltophilum*, and decreased abundance of bacteria commonly associated with oral health, *e.g. Streptococcus* spp., *Actinomyces* spp., *Gemella* spp., *Granulicatella* spp., and *Veillonella* spp.	
**44 HNSCC patients**	Oral rinses	16S rRNA sequencing	Different microbial profiles in responders and non-responders to induction CHT (TPF); abundance of *Slackia* spp. was enriched in responders; abundance of *Mycoplasma* spp. and *F. nucleatum* were enriched among non-responders	Rui *et al.* (2021) [154]
**3) Immunotherapy**				
**31 R/M HNSCC patients**	Fecal samples	16S rRNA sequencing	High gut microbial diversity is associated with better ICIs response rate; higher abundance of genus *Bacteroides* and *Lachnospiracea incerate sedis* is linked to higher response rate to ICIs; lower representation of Firmicutes was also linked to higher ICIs response rate; enriched populations of *Eu. oxidoreducens*, *Bact. uniformis,* and *Ruminococcus* spp. were observed in reponders to ICIs	Bari *et al.* (2022) [187]

**85 HNSCC patients treated with nivolumab compared to 31 treated with investigator's choice**	Unstimulated saliva	16S rRNA sequencing	Lower representation of bacterial families *Prevotellaceae* and *Flavobacteriaceae* in patients pre-treated with RT compared to RT-naive patients; no association between microbial diversity or altered bacterial representation and nivolumab treatment response	Ferris *et al*. (2022) [188]

S4: Table 4: Effect of probiotics and antibiotics on HNSCC treatment efficacy

Patients	Agent	Results / implications	Ref.
**1) Probiotics**			
**210 randomized HNSCC patients**	*Levilactobacillus brevis* CD2	Gr 3 and 4 were reported in 52% of patients receiving *Lev. brevis* CD2 lozenges compared to 77% if patients in placebo cohort; anti-cancer treatment completion was reached in 92% *vs.* 70% in study and placebo cohort, respectively; 28% *vs.* 7% patients developed no RIOM in study *vs.* placebo cohort	Sharma *et al.* (2011) [111]
**75 OSCC patients**	*Levilactobacillus brevis* CD2	No statistically significant differences in RIOM incidence and severity (40.6% *vs.* 41.6% in study and placebo cohort, respectively), no statistically significant differences in QoL	De Sanctis *et al.* (2019) [113]
**52 LA HNSCC patients**	*Levilactobacillus lactis* AG013	35% reduction of developing ulcerative RIOM (*i.e.* Gr 2 and higher) in study cohort compared to placebo; all patients in the placebo cohort had Gr 2 and higher RIOM lasting for more than 2 days; in study cohort 29% of patients had Gr 2 and higher RIOM lasting less than 2 days, in study cohort less unplanned and/or emergency visits were observed compared to the placebo cohort	Limaye *et al.* (2013) [112]
**99 LA nasopharyngeal cancer patients**	Bifico combination (*B. longum*, *Lactococcus lactis*, and *Ent. faecium*)	Patients in study cohort developed significantly less and less severe RIOM compared to placebo cohort; Gr 0, 1, 2, and 3 RIOM was observed in 0%, 0%, 17.24% and 15.52% *vs.* In 12.07%, 55.17%, 54.29% and 45.71% in study *vs.* placebo cohort, respectively	Jiang *et al.* (2019) [114]
**HNSCC cellular line (SCC15, CAL-27, WSU-HN6)**	*Bifidobacterium breve* lw01	*B. breve* lw01 produces EPS showing anti-cancer activity against HNSCC cell lines; EPS regulates cell cycle and promotes apoptosis	Wang *et al.* (2019) [117]
**2) Antibiotics**			
**220 LA HNSCC patients, 154 were**	ATB according to the investigator's choice	ATB prescriptions were done regardless of sex, age, performance status, tumor localization; ATB prescription was	Rühle *et al.* (2023) [118]

**(pre-)treated with ATB**		higher in higher tumor stages; pre-therapeutic ATB treatment did not affect PFS; peri-therapeutic ATB treatment statistically significantly diminished PFS and OS	
**272 LA HNSCC, 124 treated with antibiotics during week 1 or 2 of CRT**	ATB according to the investigator's choice	Patients treated within the week 1 or 2 had significantly lower OS and disease-specific survival; ATB administration was independently associated with reduced PFS; negative impact on patients treated with two or more courses of ATB was even greater	Nenclares *et al.* (2020) [120]
